# Associations of sleep quality and sleep duration with frailty and pre-frailty in an elderly population Rugao longevity and ageing study

**DOI:** 10.1186/s12877-019-1407-5

**Published:** 2020-01-06

**Authors:** Xue-Hui Sun, Teng Ma, Shun Yao, Ze-Kun Chen, Wen-Dong Xu, Xiao-Yan Jiang, Xiao-Feng Wang

**Affiliations:** 1 Ministry of Education Key Laboratory of Contemporary Anthropology, School of Life Sciences, Fudan University, Shanghai, China; 2 Human Phenome Institute, Fudan University, Shanghai, China; 3 National Clinical Research Center for Aging and Medicine, Huashan Hospital, Fudan University, Shanghai, China; 4 Key Laboratory of Arrhythmias of the Ministry of Education of China, Tongji University School of Medicine, Shanghai, 200092 People’s Republic of China; 5 Shanghai Key Laboratory of Clinical Geriatric Medicine and Huadong Hospital Clinical Research Center for Geriatric Medicine, Shanghai, China; 6 Unit of epidemiology, Human Phenome Institute, State Key Laboratory of Genetic Engineering and MOE Key Laboratory of Contemporary Anthropology, Fudan University, Shanghai, 200433 China

**Keywords:** Frailty, Sleep disturbances, Sleep quality, Sleep duration, Elderly population

## Abstract

**Background:**

Previous studies suggest that poor sleep quality or abnormal sleep duration may be associated with frailty. Here we test the associations of sleep disturbances with both frailty and pre-frailty in an elderly population.

**Methods:**

Participants included 1726 community-dwelling elders aged 70–87 years. Pittsburgh Sleep Quality Index (PSQI) was used to assess sleep disturbances. Frailty was defined using phenotype criteria. Logistic regression models were used to estimate odds ratio of the associations.

**Results:**

The average PSQI score was 5.4 (SD, 3.1). Overall 43.6% of the participants had poor sleep quality (PSQI> 5), 8.2% had night sleep time ≤ 5 h, and 27.8% had night sleep time ≥ 9 h. The prevalence of frailty and pre-frailty was 9.2 and 52.8%, respectively. The proportions of PSQI> 5 increased with the severity of frailty status (robust: pre-frail: frail, 34.5%: 48%: 56.1%, *P* < 0.001). After adjustment for multiple potential confounders, poor sleep quality (PSQI> 5) was associated with higher odds of frailty (OR = 1.78, 95% CI 1.19–2.66) and pre-frailty (OR = 1.51, 95% CI 1.20–1.90). Sleep latency, sleep disturbance, and daytime dysfunction components of PSQI measurements were also associated with frailty and pre-frailty. In addition, sleep time 9 h/night was associated with higher odds of frailty and pre-frailty.

**Conclusions:**

We provided preliminary evidences that poor sleep quality and prolonged sleep duration were associated with being frailty and pre-frailty in an elderly population aged 70–87 years. The associations need to be validated in other elderly populations.

## Background

Frailty is a clinical syndrome characterized by loss of physiologic reserve and resistance to stressors due to cumulative declines across multiple physiologic systems. Frailty results in vulnerability to adverse outcomes including restricted mobility, reduced self-reliance and disability, falls, hospitalization, and mortality [1–3]. Frailty is a public health problem with a prevalence of about 10% in the community-dwelling elderly population [4]. However, frailty and pre-frailty are reversible conditions if appropriately treated. Therefore, identifying high risk elders of frailty for specific management of underlining causes is important to prevent from developing to more advanced condition, such as disability. Several risk factors of frailty had been proposed and some had been validated, such as age, low socioeconomic status, cognitive impairment, and diabetes [5, 6].

Sleep disturbances are common and serious problems of the elderly population [7, 8]. About 50% of the elderly people suffers from sleep problems [7]. Sleep is extremely important to health since human body carries out a series of biological and physiological activities during sleep process, such as hormonal release, energy metabolism, glucose and cardiovascular regulation, and self-regulation and recovery of physiological functions [9]. Sleep disorders were found to be associated with increased risks of different adverse outcomes, such as obesity, hypertension, cognitive impairment, depression, and death [10–14].

Recently, poor sleep quality was reported to be associated with prevalent [15, 16] and incident frailty at follow-up in U.S. community-dwelling elders [17]. In addition, an U-shaped sleep duration-frailty association [17, 18] or prolonged sleep duration-frailty relationship [19, 20] was also reported to community dwellers. However, more evidences need to be accumulated before sleep disturbances established as a behavior risk factors of frailty.

In this study, we aimed to investigate the associations of different symptoms of sleep disturbance (poor sleep quality, several components of sleep quality, prolonged and insufficient sleep duration) with frailty in a community-dwelling population aged 70–87 years.

## Methods

### Study design and participants

We used data of the ageing arm of the Rugao Longevity and Ageing Study (RuLAS), a population-based observational two-arm cohort study conducted in Rugao, a typical, medium-sized city of Jiangsu province, China. The ageing arm of RuLAS is a longitudinal cohort followed-up every 1.5 years. A detailed description of this study was provided elsewhere [21]. Briefly, we recruited 1788 elders from 31 rural communities of Jiang’an Township of Rugao city between in Nov-Dec. 2014 (wave1) according to 5-year age and sex strata. From Apri. 2016 through Jun. 2016, we conducted the second-wave examination of the ageing arm, excluding 297 subjects (55 died and 242 did not come) and including an additional 333 subjects. Among the participants in the second wave, 1726 subjects aged 70–87 years with complete data on sleep and frailty variables were included in the present study. During the fieldwork, all participants were taken a fasting blood sample drawn. Then they participated in a face-to-face interview and physical examination. All of them answered a structured questionnaire themselves. This study was approved by the Human Ethics Committee of the School of Life Sciences of Fudan University, Shanghai, China. Written informed consents were obtained from all the participants.

### Sleep measurements

The participants finished the Pittsburgh Sleep Quality Index (PSQI) at wave 2 survey.The PSQI questionnaire consists of 19 items which include components of sleep quality, sleep latency, sleep duration, sleep efficiency, sleep disturbances, sleep medication use, and daytime dysfunction [22]. Each component is coded on a 4-point scale (0–3), with high scores reflecting more sleep symptoms. The Chinese version of the PSQI have good overall reliability (*r* = 0.82–0.83) and test-retest reliability (*r* = 0.77–0.85) in community adults with primary insomnia [23]. Global scores provide an assessment of overall sleep quality which ranges from 0 to 21, with scores of > 5 indicating poor sleep quality [23].

### Frailty measurements

The widely used Fried’s phenotype criterion includes unintentional weight loss, weakness, exhaustion, slowness, and low activity components [24]. We used a similar definition to measure frailty phenotype [25, 26]. In brief, unintentional weight loss, exhaustion, and low activity were measured with self-reported items “weight has decreased by 4.5 kg or 5% during the last 12 months”, “energetic most of the time or feeling tired all of the time (at least 3 or 4 days a week)”, and “needing help to walk”, respectively. Slowness was defined as being below the 20th sex-specific percentile in gait speeds measured with a timed ‘up and go’ (TUG) test (the time stand up from an armchair, walk 3 m, return, and sit down again). Weakness was defined as being below the 20th sex-specific percentile in maximum handgrip strength. Grip strength in kilograms was measured using a dynamometer (Shanghai Wanqing Rlrctron Co. Ltd., Shanghai, China) for three attempts of each hand. The max value of two hands was used in this study. Participants with any three or more indicators is defined as “frail”, any one or two is pre-frail, and zero is“non-frail”.

### Covariates

Covariates included age (70–74 years, 75–79 years, 80–84 years, 85+ years), gender (men; women), occupation (farmers, others), marital status (currently married, others), education level (illiterate; literate), smoking (none; ever, current smoker), drinking status (none, ever, current drinker), perceived overall health status (good, poor), and body mass index (BMI, < 24 kg/m^2^, 24 to 27.9 kg/m^2^, or ≥ 28 kg/m^2^) [27]. Diabetes was defined as having a diabetic history, on any anti-diabetic agent, or a fasting plasma glucose level of> 7.0 mmol/L.

Hypertension was defined as having a hypertension history, or a mean blood pressure higher than 140/90 mmHg, or on any antihypertensive agent. The Hasegawa Dementia Scale-Revised (HDS-R), including 11 items to measure orientation, memory, attention/calculation and verbal fluency, is a widely used brief and reliable measurement for evaluating global cognitive function [28, 29]. The presence of mild cognitive impairment (MCI) was defined by a HDS-R of ≤21.5 [30].

### Statistical analysis

Descriptive statistics were used as percentages or mean ± standard deviation (SD). The Chi-square test was used for comparison of categorical variables among frail groups. Logistic regression models were used to estimate odds ratios and 95% confidence intervals for the associations of sleep variables with frailty. Potential confounding factors adjusted including age, gender, occupation, marital status, education level, smoking status, drinking status, BMI category, hypertension, diabetes, MCI, and perceived overall health. Statistical analyses were performed with IBM SPSS 19.0 (IBM Corporation, Armonk, NY, USA). A two-sided *p* < 0.05 was considered significant.

## Results

### Characteristics of the study population

The average PSQI score of the studied elderly population (aged 77.6 ± 3.9 years) was 5.4 (SD, 3.1) and the average night sleep time 7.6 h (SD, 1.7 h). Overall 43.6% of the elderly adults had poor sleep quality (PSQI> 5), 8.2% had night sleep time ≤ 5 h, and 27.8% had night sleep time ≥ 9 h. The prevalence of frailty and pre-frailty was 9.2 and 52.8%, respectively. The percentages of female, illiterate, uncoupling, diabetes, MCI decreased from robust to pre-frail, then to frailty groups while the percentages of smokers and alcohol drinkers increased (*p* < 0.05) (Table 1).

**Table 1:** Demographic characteristics of participants by frailty status

characteristics	All (*n* = 1726)	Robust (*n* = 656)	Pre-frail (*n* = 911)	Frail (*n* = 159)	*P*_value
Age groups (years)					< 0.001
70–74	427 (24.7)	204 (31.1)	204 (22.4)	19 (11.9)	
75–79	773 (44.8)	314 (47.9)	402 (44.1)	57 (35.8)	
80–84	415 (24)	120 (18.3)	235 (25.8)	60 (37.7)	
85^+^	111 (6.4)	18 (2.7)	70 (7.7)	23 (14.5)	
Gender					< 0.001
Male	816 (47.3)	385 (58.7)	382 (42)	49 (30.8)	
Female	909 (52.7)	271 (41.3)	528 (58)	110 (69.2)	
Occupation					0.032
Farmer	1502 (88.6)	566 (87.6)	790 (88.6)	146 (93)	
Other	193 (11.4)	80 (12.4)	102 (11.4)	11 (7)	
Education					< 0.001
Illiterate	885 (51.6)	260 (39.9)	515 (57)	110 (69.2)	
Literate	829 (48.4)	391 (60.1)	389 (43)	49 (30.8)	
Marital status					< 0.001
Current marital	1126 (66)	459 (70.8)	585 (65)	82 (52.2)	
Other	579 (34)	189 (29.2)	315 (35)	75 (47.8)	
Smoking					< 0.001
None	1251 (73.7)	427 (65.8)	696 (77.9)	128 (82.1)	
Smoker	301 (17.7)	159 (24.5)	123 (13.8)	19 (12.2)	
Ever	146 (8.6)	63 (9.7)	74 (8.3)	9 (5.8)	
Drinking					< 0.001
None	1098 (65.1)	364 (56.7)	617 (69.1)	117 (76.5)	
Drinker	443 (26.2)	218 (34)	199 (22.3)	26 (17)	
Ever	147 (8.7)	60 (9.4)	77 (8.6)	10 (6.5)	
BMI category					0.163
< 24	931 (54.9)	334 (51.9)	509 (56.8)	88 (56.1)	
24–27.9	574 (33.8)	240 (37.3)	291 (32.5)	43 (27.4)	
≥ 28	192 (11.3)	70 (10.9)	96 (10.7)	26 (16.6)	
Perceived overall health status					0.448
Good	1432 (83.6)	600 (92)	738 (81.6)	94 (59.5)	
Poor	282 (16.5)	52 (8)	166 (18.4)	64 (40.5)	
Hypertension					0.171
No	436 (25.5)	176 (27.2)	221 (24.4)	39 (24.5)	
Yes	1277 (74.6)	472 (72.8)	685 (75.6)	120 (75.5)	
Diabetes					< 0.001
No	1401 (82.3)	545 (84.4)	734 (81.3)	122 (79.2)	
Yes	302 (17.7)	101 (15.6)	169 (18.7)	32 (20.8)	
MCI					< 0.001
No	889 (51.7)	438 (67)	408 (44.8)	43 (27.4)	
Yes	832 (48.3)	216 (33)	502 (55.2)	114 (72.6)	

### Associations of sleep quality with frailty

The proportions of participants with poor sleep quality (PSQI> 5) increased with the severity of frailty in the elderly (robust: pre-frail: frail, 34.5%: 48%: 56.1%, *P* < 0.001) (Table 2).

**Table 2:** Distribution and comparison of the PSQI scores among robust, pre-frail and frail groups

Sleep variables	Robust(656)	Pre-frail(911)	Frail(157)	*P*
Poor subjective sleep quality				< 0.001
Total score ≤ 5	430 (65.5)	473 (52)	69 (43.9)	
Total score > 5	226 (34.5)	436 (48)	88 (56.1)	
Sleep quality component				< 0.001
0	155 (23.7)	212 (23.5)	25 (15.9)	
1	427 (65.4)	524 (58)	95 (60.5)	
2	64 (9.8)	156 (17.3)	33 (21)	
3	7 (1.1)	12 (1.3)	4 (2.5)	
Sleep latency component				< 0.001
0	231 (36.9)	234 (27.4)	40 (27.6)	
1	276 (44.1)	396 (46.4)	50 (34.5)	
2	81 (12.9)	137 (16)	30 (20.7)	
3	38 (6.1)	87 (10.2)	25 (17.2)	
Sleep duration component				0.021
0	342 (53.3)	482 (54.2)	90 (59.6)	
1	244 (38)	329 (37)	37 (24.5)	
2	31 (4.8)	46 (5.2)	13 (8.6)	
3	25 (3.9)	33 (3.7)	11 (7.3)	
Sleep efficiency component				0.002
0	322 (50.5)	409 (47.3)	65 (43.6)	
1	129 (20.2)	149 (17.2)	23 (15.4)	
2	76 (11.9)	123 (14.2)	13 (8.7)	
3	111 (17.4)	184 (21.3)	48 (32.2)	
Sleep disturbances component				< 0.001
0	31 (4.7)	24 (2.6)	2 (1.3)	
1	531 (80.9)	640 (70.3)	102 (65)	
2	93 (14.2)	242 (26.6)	51 (32.5)	
3	1 (0.2)	4 (0.4)	2 (1.3)	
Sleep medication use component				0.147
0	643 (98.8)	888 (98.4)	149 (96.8)	
1	2 (0.3)	1 (0.1)	2 (1.3)	
2	4 (0.6)	5 (0.6)	2 (1.3)	
3	2 (0.3)	8 (0.9)	1 (0.6)	
Daytime dysfunction component				< 0.001
0	465 (71.6)	469 (51.8)	54 (34.4)	
1	120 (18.5)	176 (19.4)	40 (25.5)	
2	59 (9.1)	203 (22.4)	37 (23.6)	
3	5 (0.8)	57 (6.3)	26 (16.6)	–

After adjustment for multiple potential confounders including age group, gender, occupation, education, smoking, drinking, BMI category, diabetes, hypertension, MCI, and perceived overall health status, poor sleep quality (PSQI> 5) was associated with higher odds of being frailty (OR = 1.78, 95% CI 1.19–2.66). With 1 point increments in PSQI score, the odds ratio of frailty increased by 16% (OR = 1.16, 95% CI (1.09–1.23). Poor sleep quality defined by PSQI> 5 was also significantly associated with the odds of pre-frailty compared with the robust participants independent of other factors pre-frailty (OR = 1.51, 95% CI 1.20–1.90). With per SD increments in PSQI score, the multivariable-adjusted odds of pre-frailty increased by 11%, OR = 1.11, 95% CI (1.04–1.13) (Table 3). In addition, using PSQI> 6 as cut-point [23], we also found significant associations of poor sleep quality with increased odds of pre-frailty and frailty (data not shown).

**Table 3:** Associations between sleep quality and components of sleep quality and frailty status by logistic regression analysis

Sleep quality	Crude model	Model1	Model2	Model3
Pre-frail				
Poor subjective sleep quality	1.75 (1.43–2.16)***	1.61 (1.3–1.99)***	1.63 (1.3–2.04)***	1.51 (1.20–1.90)***
PSQI> 5 vs. ≤ 5				
PSQI global score	1.11 (1.08–1.15)***	1.1 (1.06–1.14)***	1.1 (1.06–1.14)***	1.08 (1.04–1.13)***
Per SD PSQI score	1.41 (1.26–1.57)***	1.35 (1.21–1.52)***	1.35 (1.2–1.52)***	1.29 (1.14–1.46)***
Sleep quality component	1.22 (1.04–1.42)*	1.15 (0.98–1.36)	1.15 (0.97–1.37)	1.08 (0.91–1.29)
Sleep latency component	1.3 (1.16–1.46)***	1.24 (1.1–1.4)***	1.24 (1.1–1.41)**	1.23 (1.08–1.4)**
Sleep duration component	0.98 (0.86–1.12)	0.95 (0.83–1.09)	0.93 (0.8–1.07)	0.9 (0.78–1.05)
Sleep efficiency component	1.1 (1.01–1.2)*	1.05 (0.96–1.15)	1.05 (0.96–1.16)	1.01 (0.92–1.12)
Sleep disturbance component	2.01 (1.6–2.51)***	1.97 (1.57–2.48)***	2.04 (1.6–2.59)***	1.88 (1.46–2.4)***
Sleep medication use component	1.21 (0.82–1.77)	1.17 (0.79–1.73)	1.01 (0.67–1.52)	1.01 (0.65–1.56)
Daytime dysfunction component	1.85 (1.62–2.1)***	1.9 (1.66–2.17)***	1.9 (1.65–2.19)***	1.9 (1.64–2.2)***
Frail				
Poor subjective sleep quality	2.43 (1.7–3.46)***	2.15 (1.49–3.09)***	2.19 (1.49–3.21)***	1.78 (1.19–2.66)**
PSQI> 5 vs. ≤ 5				
PSQI global score	1.22 (1.16–1.29)***	1.2 (1.14–1.27)***	1.2 (1.13–1.27)***	1.16 (1.09–1.23)***
Per SD PSQI score	1.87 (1.58–2.21)***	1.77 (1.49–2.11)***	1.75 (1.46–2.1)***	1.6 (1.32–1.94)***
Sleep quality component	1.66 (1.28–2.16)***	1.54 (1.17–2.01)**	1.51 (1.14–2)**	1.24 (0.92–1.68)
Sleep latency component	1.6 (1.32–1.93)***	1.5 (1.23–1.83)***	1.45 (1.18–1.79)***	1.39 (1.12–1.72)**
Sleep duration component	1.07 (0.86–1.34)	1.02 (0.81–1.27)	1.02 (0.8–1.28)	0.95 (0.74–1.23)
Sleep efficiency component	1.25 (1.08–1.45)**	1.16 (1–1.34)	1.16 (0.99–1.35)	1.08 (0.91–1.27)
Sleep disturbance component	2.84 (1.99–4.05)***	2.84 (1.97–4.1)***	2.85 (1.94–4.19)***	2.34 (1.55–3.51)***
Sleep medication use component	1.43 (0.85–2.41)	1.34 (0.78–2.29)	1.21 (0.68–2.17)	1.16 (0.62–2.19)
Daytime dysfunction component	2.67 (2.22–3.22)***	2.85 (2.34–3.46)***	2.9 (2.36–3.55)***	2.8 (2.25–3.48)***

Model1 adjusted for age, and gender on crude model. Model2 adjusted for smoking, drinking, education, marital status, occupation, BMI category plus the variables in Model1. Model3 adjusted for diabetes, hypertension, MCI, perceived overall health plus the variables in Model 2

*P < 0.05, **P < 0.01, ***P < 0.001

### Associations of sleep quality component with frailty

For components of PSQI, on the whole, higher percentages of sleep symptoms were found in sleep quality component, sleep latency component, sleep duration component, sleep efficiency component, sleep disturbance component, and daytime dysfunction component from robust group to pre-frail group, and then to frail group (Table 2). After adjustments for multiple covariates, sleep latency, sleep disturbance, and daytime dysfunction components were significantly associated with higher odds of pre-frailty and frailty (Table 3).

#### Associations of sleep duration with frailty

Table 4 showed the associations between reported sleep duration and frailty. The odds of being frail in those with a sleep time ≤ 5 h per night was significantly increased compared to those with a sleep time 7–8 h. However, the OR attenuated with the addition of adjustment variables and was not statistically significant after adjustment for sleep quality.

**Table 4:** Associations between sleep duration and frailty status by logistic regression analysis

Sleep duration (h per 24-h period)	Crude model	Model 1	Model 2	Model 3
Pre-frail				
≤ 5(*n* = 137)	0.9 (0.58–1.4)	1.04 (0.68–1.6)	1.11 (0.74–1.68)	1.31 (0.87–1.95)
5–7 (*n* = 360)	1.29 (0.97–1.73)	1.33 (1–1.77)	1.3 (1–1.71)	1.36 (1.04–1.77)*
7–8 (*n* = 712)	1	1	1	1
≥ 9 (*n* = 466)	1.82 (1.37–2.42)***	1.86 (1.41–2.45)***	1.84 (1.41–2.4)***	1.96 (1.51–2.53)***
Frail				
≤ 5(*n* = 137)	2.93 (1.63–5.27)***	2.04 (1.09–3.81)*	1.95 (1.01–3.77)*	1.77 (0.84–3.73)
5–7 (*n* = 360)	0.78 (0.43–1.39)	0.69 (0.38–1.27)	0.75 (0.4–1.38)	0.64 (0.32–1.27)
7–8 (*n* = 712)	1	1	1	1
≥ 9 (*n* = 466)	2.98 (1.95–4.56)***	2.4 (1.53–3.77)***	2.43 (1.51–3.91)***	2.55 (1.49–4.35)**

Model1 adjusted for age, and gender on crude model. Model2 adjusted for smoking, drinking, education, marital status, occupation, BMI category, diabetes, hypertension, MCI, perceived overall health plus the variables in Model 1. Model3 further added sleep quality (P PSQI> 5 vs. ≤ 5) to model 2

*P < 0.05, **P < 0.01, ***P < 0.001

Long sleep duration of ≥9 h, compared with sleep 7-8 h, was associated with increased risk of pre-frailty (OR = 1.82, 95% CI 1.37–2.42) and frailty (OR = 2.98, 95% CI 1.95–4.56), and the correlation was not affected by other factors such as sleep quality (Table 4).

## Discussion

In the present study, we found that poor sleep quality, long sleep latency, sleep disturbance, daytime dysfunction, and longed sleep duration were associated with increased odds of frailty in an elderly population. In addition, to the best of our knowledge, for the first time, we found that sleep disturbance variables such as sleep latency, were associated pre-frailty in this elderly population aged 70–87 years.

In the Osteoporotic Fractures in Men (MrOS) cohort of U.S. males aged 67 years and older, Ensrud [15] et al. found that poor subjective sleep quality of PSQI> 5, objective parameters of less sleep efficiency, and objective parameters of sleep disordered breathing were independently associated with a higher odd of being frail in cross-sectional analysis. Later, in a prospective study follow-up of an average of 3.4 years, they replicated these associations [17]. Our results are similar to their observations that poor subjective sleep quality was associated with frailty status in Rugao population. In addition, we extended the associations to pre-frailty status which is even more clinically relevant since it is reversible condition when timely intervened.

Poor sleep quality measured by PSQI was associated with frailty not only in community population setting but also in institutionalized setting where frail elderly (374 elderly residents of long-stay institutions aged 77.52 ± 7.82 years) exhibited poor sleep quality [31]. In addition, the associations were also observed for frailty measured with other instruments. In 351 Atahualpa residents aged ≥60 years, Brutto et al. found that higher scores of the PSQI were significantly associated with higher scores in the Edmonton Frail Scale [16].

For the component of sleep quality, we found that long sleep latency and sleep disturbances were associated with increased risks of pre-frailty and frailty in Rugao population. This is similar to the observations that long sleep latency and poor sleep efficiency measured by objective actigraph were associated with higher odds of prevalent frailty [15] and higher risk of incident frailty [17]. Another significant component associated with frailty we observed in this population aged 70–87 years is daytime dysfunction. To the best of our knowledge, this is the first report correlated daytime dysfunction with frailty status in elderly population since other studies such as Ensrud et al. [15, 17] did not specially analyze the relationship between PSQI components and frailty. However, using the objective actigraph measurements, Ensrud et al. found that another relevant symptoms, excessive daytime sleepiness, were associated with increased odds of frailty in the MrOS cohort [15]. This corroborates our observations from another perspective.

The observations addressing the relationship between sleep duration and frailty were less consistent. Both long sleep duration (≥9 h) and short sleep duration (≤6 h or ≤ 5 h) were associated with higher odds of frailty in Japanese [18] and Danish population [32]. However, only long sleep duration (≥10 h or ≥ 9 h or ≥ 8 h) was found associated with higher odds of frailty in American elderly [19], our Chinese elderly, or Korean elderly people [20], respectively. Interestingly, using data of NHANES cohort of U.S. population, Zhang et al. found that sleeping > 9 h was associated with frailty in males and sleeping < 6 h were associated with frailty in females, which indicate a gender specific association [33]. Low levels of daily exercise, low muscle strength, and very slow walking speeds, all indicative of prefrailty/frailty also showed prolonged sleep duration (≥9 h) in a British elderly population [34]. Since both prolonged sleeping hours and frailty may be manifestations or biomarkers of disease status of the studied participant, in the present study, we adjusted several health variables in the analysis models and still observed the associations. In addition, since elderly people may spend more time in bed when they are frail (i.e. reverse causality), and aforementioned cross-sectional data cannot provide evidence for the direction of the association, evidences in the prospective studies need to be accumulated with respect to the association between sleep duration and frailty.

Several possible underline mechanisms may help explain the associations of sleep disturbances with frailty. (1) sleep symptoms may be markers for comorbidities and poor health which increases the likelihood of frailty [17]. Although we adjusted multiple disease covariates in the present study, we could not exclude this possibility. (2) The disrupted circadian rhythms induced by sleep disorder may contribute to dysregulation of immune system with increased systemic inflammation factors which contribute to the development of frailty [35]. (3) sleep disorder may contribute to increased oxidative stress and alterations of metabolic pathways favoring catabolism, which could serve as a combined risk for the development of frailty [36]. (4) Disturbed sleep may reduce growth hormone, insulin-like growth factor-1, and sex hormone such as testosterone secretion, which in turn enhance muscle proteolysis, thus, leading to sarcopenia and frailty [37]. In the future study, we need to measure these cytokines in our participants to explore whether they mediate the sleep-frailty associations.

The limitations of this study need to be mentioned. The cross-sectional nature of this study prohibits causal inference since the relationship between frailty and sleep disturbances may be bidirectional. In addition, it’s a pity that we did not measure the actigraphic or polysomnographic parameters in our cohort, therefore, we could not validate the objective associations of sleep disturbances and sleep disordered breathing with frailty. Further, a significantly large percentage of the subjects were farmers, such a specific occupation is not only physically demanding that could impact frailty but also have a particularly unique regimental challenges to one’s sleep. This may affect the generalizability of our findings.

## Conclusions

In summary, we found that poor subjective sleep quality, some sleep symptoms measured by PSQI, and prolonged sleep duration were associated with higher odds of frailty, and even pre-frailty in an elderly population aged 70–84 years However, since the associations were cross-sectional, the effects of sleep disturbances on frailty incidences need to be validated in the prospective cohort studies of the elderly population of this age group.

## Abbreviations

BMI: Body mass index
CRP: C reactive protein
DBP: Diastolic blood pressure
HDS: Hasegawa Dementia Scale
HDS-R: Hasegawa Dementia Scale-Revised
MCI: Mild cognitive impairment
MrOS: Osteoporotic Fractures in Men
NHANES: The National Health and Nutrition Examination Survey
OR: Odds ratio
PSQI: Pittsburgh Sleep Quality Index
RuLAS: Rugao Longevity and Ageing Study
SBP: Systolic blood pressure
TUG: Up and go
